# Understanding the complex determinants of height and adiposity in disadvantaged daycare preschoolers in Salvador, NE Brazil through structural equation modelling

**DOI:** 10.1186/s12889-015-2406-x

**Published:** 2015-10-23

**Authors:** Rebecca L. Lander, Sheila M. Williams, Hugo Costa-Ribeiro, Angela P. Mattos, Danile L. Barreto, Lisa A. Houghton, Karl B. Bailey, Alastair G. Lander, Rosalind S. Gibson

**Affiliations:** Department of Human Nutrition, University of Otago, PO Box 56, Dunedin, 9015 New Zealand; Department of Preventive and Social Medicine, University of Otago, PO Box 56, Dunedin, New Zealand; Hospital Universitario Professor Edgard Santos, Fima Lifshitz Research Unit, Salvador, Bahia Brazil

**Keywords:** Structural equation modelling, Height, Body mass index, Adiposity, Disadvantaged preschoolers

## Abstract

**Background:**

Earlier we reported on growth and adiposity in a cross-sectional study of disadvantaged Brazilian preschoolers. Here we extend the work on these children, using structural equation modelling (SEM) to gather information on the complex relationships between the variables influencing height and adiposity. We hope this information will help improve the design and effectiveness of future interventions for preschoolers.

**Methods:**

In 376 preschoolers aged 3–6 years attending seven philanthropic daycares in Salvador, we used SEM to examine direct and indirect relationships among biological (sex, ethnicity, birth order, maternal height and weight), socio-economic, micronutrient (haemoglobin, serum selenium and zinc), and environmental (helminths, de-worming) variables on height and adiposity, as reflected by Z-scores for height-for-age (HAZ) and body mass index (BMIZ).

**Results:**

Of the children, 11 % had HAZ < −1, 15 % had WHZ < −1, and 14 % had BMIZ > 1. Of their mothers, 8 % had short stature, and 50 % were overweight or obese. Based on standardized regression coefficients, significant direct effects (*p* < 0.05) for HAZ were maternal height (0.39), being white (−0.07), having helminth infection (−0.09), and serum zinc (−0.11). For BMIZ, significant direct effects were maternal weight (0.21), extremely low SES (−0.15), and haemoglobin (0.14). Indirect (*p* < 0.05) effects for HAZ were sex (being male) (−0.02), helminth infection (−0.01), de-worming treatment (0.01), and serum selenium (−0.02), and for BMIZ were extremely low SES (−0.001), helminth infection (−0.004), and serum selenium (0.02).

**Conclusions:**

Of the multiple factors influencing preschoolers’ growth, helminth infection was a modifiable risk factor directly and indirectly affecting HAZ and BMIZ, respectively. Hence the WHO de-worming recommendation should include preschoolers living in at-risk environments as well as school-aged children.

## Background

Inter-related pathways with the potential to lead to malnutrition among children in disadvantaged settings are complex, as highlighted by UNICEF in a revised conceptual model [1]. Such complex pathways almost certainly exist in the urban slums of Salvador, NE Brazil, where malnutrition among disadvantaged preschoolers was characterized by co-existing impaired height and overweight [2]. This double burden of malnutrition has been reported elsewhere in Brazil [3] and in other countries in the midst of a rapid nutrition transition [4].

Several adverse health consequences are associated with impaired linear growth and overweight in early childhood. Impaired linear growth is associated with impairments in cognitive and motor development [5] that may persist into adulthood [6]. In contrast, overweight children are at high risk of adolescent and adult obesity and associated type 2 diabetes, hyperlipidemia, and hypertension [7, 8]. There is also mounting evidence that stunting may predispose children in later life to the same risks as overweight and obesity in childhood [9, 10].

Intestinal parasitic infections have also been linked to impaired linear growth in children in NE Brazil [11] and elsewhere [12], and may be associated with obesity and its comorbidities in later life [13]. The interplay among these multiple inter-related factors has been termed the triple burden of poverty by some investigators [14]. Genetic factors also play a role in linear growth, and may be especially important in Salvador, NE Brazil where 80 % of the population is of West African descent [15].

Clearly the etiology of impaired height and overweight among disadvantaged preschool children in Brazil is complex, making the design and implementation of effective intervention strategies challenging. Here we have applied structural equation modeling (SEM) to understand the multiple factors influencing the height and adiposity of a group of disadvantaged preschoolers attending philanthropic daycare centers in Salvador, NE Brazil. Both direct and indirect relationships between socio-economic, biological, environmental, and micronutrient variables on height and adiposity, as reflected by Z-scores for height-for-age (HAZ) and body mass index (BMIZ), were examined. Variables chosen were known or hypothesized to influence HAZ and BMIZ-scores of the preschoolers based on our earlier cross-sectional findings [16, 17] and theoretical evidence from the literature. It was expected that use of the SEM would broaden our understanding of the complex inter-relationships among any modifiable variables associated with height and adiposity, and thus help in designing tailored and potentially more effective interventions for disadvantaged preschoolers in similar settings.

## Methods

### Study sites and participants

Details of the recruitment of the preschoolers, their socio-demographic, anthropometric, micronutrient, and infection status have been reported earlier [16, 17]. Briefly, a cross-sectional study of 376 disadvantaged preschoolers aged between 3 and 6 years was conducted in seven philanthropic daycare centers located in Salvador, the capital city of Bahia, NE Brazil from August – November, 2010. All seven daycares implemented standardized use of clean drinking water, hygienic sanitation, regular physical exercise programs, and maternal nutrition education, in addition to providing standardized micronutrient-rich meals which comprised the majority of the weekday food supply for the children.

Inclusion criteria were apparently healthy children from poor communities enrolled full-time (i.e. 07.30–17.00 h) in the daycare classes. Of the 438 eligible children, the caregivers of 376 children (86 %) consented to participate in the study. Of the 14 % (*n* = 62) of children excluded, 26 % (*n* = 16) moved during the data collection, 5 % (*n* = 3) were chronically ill, and 69 % (*n* = 43) were not allowed to participate in the study. Study protocols were approved by the responsible philanthropic organizations and the Human Ethics Committees of the Federal University of Bahia, Salvador and the University of Otago, New Zealand. Informed written consent was given by the parents or guardians of the children.

### Assessment of socio-economic and biological variables

Information on socio-economic status (SES), birth order, and ethnicity of the preschoolers was collected from maternal reports using an interviewer-administered questionnaire. An overall SES score was developed for each child based on data on parental education and occupation, sanitation, ownership of possession, and other household characteristics; details are given elsewhere [16]. Ethnicity of the children was determined by skin colour, hair and facial characteristics [15]. Height and weight of the mothers and their children were measured using standardized techniques and calibrated equipment. Mothers with height < 150 cm were classified as short [18]. For the children, Z-scores for height-for-age (HAZ), weight-for-height (WHZ) and BMI were calculated using the WHO 2006/2007 growth reference data [19, 20]. Children were classified as stunted or mildly stunted, and wasted and mildly wasted based on HAZ- and WHZ-scores of < −2 or < −1 to ≥ −2SD, respectively. Overweight or obesity among the children was based on BMIZ > 1 to ≤ 2SD or > 2SD, respectively. Mothers with a BMI < 18.5 were classified as under-weight, and those with a BMI ≥ 25 or BMI ≥ 30 as overweight or obese, respectively [21].

### Assessment of environmental variables and micronutrient status

A faecal concentrate was examined by microscopy for the presence of helminths and hookworm, whereas data on the treatment for de-worming in the last 6 months was obtained by mother or caregiver report via questionnaire [16]. Morning fasting veni-puncture blood samples were drawn for analyses of haemoglobin (Hb), serum zinc and selenium, as described in detail earlier [17]. Briefly, serum zinc was analyzed by flame atomic absorption spectrophotometry (ContrAA 700, Analytik Jena, Germany) and serum selenium by electrothermal atomic absorption spectrophotometry (AA-800, Perkin Elmer 2690, Ebos Group Ltd, Auckland, New Zealand). The between-assay coefficients of variation (CV as %) for serum zinc and selenium were 5 and 7 %, respectively and the values for the certified reference materials fell within the certified ranges. Anaemia was defined as Hb < 110 g/L and < 115 g/L for children < 5 years and ≥ 5 years, respectively, and low zinc and selenium status as serum zinc < 9.9 μmol/L and serum selenium ≤ 0.82 μmol/L, respectively [17].

### Statistical analysis

**Fig. 1:**
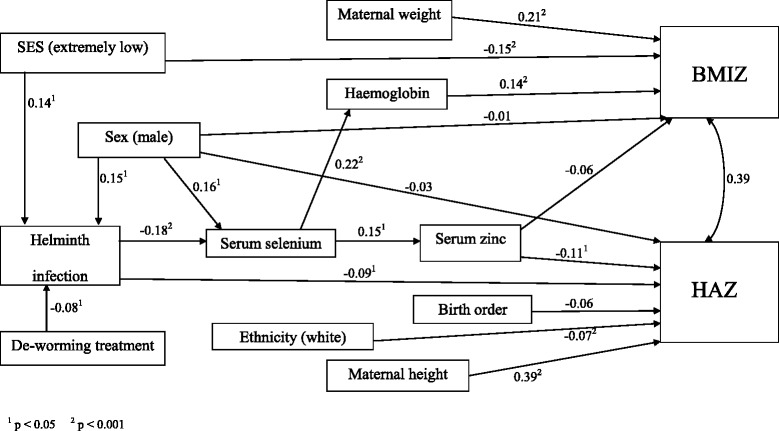
Structural equation model for adiposity and growth with outcomes BMIZ and HAZ

A correlation matrix was generated using Pearson’s correlation coefficients for each of the variables known or hypothesized to influence HAZ and BMIZ of the preschoolers, based on our earlier cross-sectional findings [12, 18] and theoretical evidence from the literature. Dietary intake was not included as a variable because only data on the nutrients supplied from the daycare menus were collected and not the usual nutrient intakes of each child. A conceptual model showing each known or hypothesised causal path relationship between the chosen variables and HAZ and BMIZ was explored using SEM. Maximum-likelihood with missing values (MLMV) was used to estimate the parameters. Both the direct and indirect path coefficients are presented by standardized regression weights (β) shown beside the arrows in Fig. 1. Standardized regression weights (β) correspond to effect size estimates and reflect the degree of change in the standard deviation of the outcome variable associated with a standard deviation change in the predictor. They compare predictor-outcome relationships across studies even though the variables have been measured using different units of measure; standardized regression weights (β) with *p* values < 0.05 were considered significant. The fit of the model to the data was tested using the coefficient of determination and the root-mean-square residual (RMSR), a value less than 0.05 reflecting good model fit. The sandwich estimator was used to obtain robust standard errors to account for the sampling procedure. Statistical analyses were carried out using STATA version 12 (Stata Corporation, College Station, TX, USA).

## Results

### Characteristics of the preschoolers and their mothers

Nearly 50 % of the preschoolers were from extremely low SES households. Most of the children were ethnically either black or brown and 33 % of high birth order (3rd or subsequent birth). No difference existed between SES and child ethnicity (Table 1). Nearly 20 % of the children had a helminth infection, even though approximately 50 % had received de-worming treatment within the past six months. Very few children were anaemic or had low zinc status; ~10 % had low selenium status (Table 1).

**Table 1:** Socio-demographic characteristics and prevalence of anaemia, low zinc and selenium status of the preschool children

	Number	Percent
Sex (male)	196/376	52.1
Ethnicity		
Black	154/365	42.2
Brown	189/365	51.8
White	22/365	6.0
Birth order (3rd or subsequent)	127/376	33.8
Socioeconomic status (extremely low)	182/376	48.4
Dietary supplements		
Vitamin A	204/376	54.3
Iron syrup within 6 months	200/376	53.2
Helminth infection	58/325	17.8
De-worming treatment within 6 months	192/376	51.1
Anaemia		
< 5 years (Hb < 110 g/L)	11/319	3.4
≥ 5 years (Hb < 115 g/L)	3/40	7.5
Low Zn status (serum Zn < 9.9 μmol/L)	13/358	3.6
Low Se status (serum Se ≤ 0.82 μmol/L)	34/358	9.5

Overall 11 % of the children were stunted or mildly stunted, 15 % were wasted or mildly wasted, and 14.3 % were classified as overweight or obese (Table 2). Of the mothers or primary caregivers, 53 % were overweight or obese, with less than 5 % underweight. Of the wasted or mildly wasted children (15 %), 36 % had overweight or obese mothers or caretakers, whereas for the overweight or obese children (14 %), 72 % had mothers or caretakers classified as overweight or obese.

**Table 2:** Anthropometric variables for children, their mothers, and child-mother diads

	Mean (95 % CI)	
Children		
HAZ Males	0.13 (−0.03, 0.29)	
HAZ Females	0.23 (0.07, 0.39)	
BMIZ Males	−0.33 (0.50, −0.16)	
BMIZ Females	−0.07 (−0.25, 0.11)	
	n	%
Stunted (HAZ < −2SD)	9/364	2.5
Mildly stunted (HAZ < −1 ≥ −2SD)	32/364	8.8
Wasted (WHZ < −2SD)	5/361	1.4
Mildly wasted (WHZ < −1 ≥ −2SD)	49/361	13.6
Overweight (BMIZ > 1 ≤ 2SD)	40/364	11.0
Obese (BMIZ > 2SD)	12/364	3.3
Stunted or mildly stunted & overweight or obese	7/41	17.1
Mothers		
Short (Height < 150 cm)	25/312	8.0
Underweight (BMI < 18.5)	13/312	4.2
Overweight (BMI ≥ 25)	100/312	32.1
Obese (BMI ≥ 30)	67/312	21.5
Short & overweight or obese	13/25	52.0
Child-Mother Diads		
Wasted or mildly wasted children with overweight or obese mothers	15/42	35.7
Overweight or obese children with overweight or obese mothers	33/46	71.7

### Correlation matrix of proposed variables for the SEM

The correlation matrix for the variables proposed for the SEM is shown in Table 3. Sex, ethnicity, birth order, and maternal weight and height represent unmodifiable biological variables known to affect growth [22–25]. Helminth infection, a modifiable biological variable, was also included because it has been linked with impaired growth in children [26], and was also shown earlier [17] to have an independent and negative effect on serum selenium concentrations in these preschoolers. Serum selenium was also included because it was a modest predictor of serum zinc and a strong predictor of Hb, which in turn had a positive relationship with BMIZ in our earlier univariate regression analysis [17]. Finally, because extremely low SES was negatively associated (*p* < 0.05) with BMIZ of the children and maternal body weight, but positively related to risk of helminth infection among the children, SES was also chosen as a factor in the SEM [16].

**Table 3:** Correlation matrix of biological, socio-economic, environmental, and micronutrient status variables affecting the children’s BMIZ and HAZ-scores (*n* = 376)

	Sex male	Black	White	Birth order	SES ex-low	Hel-minth	De-worm	Mat. Wt.	Mat. Ht.	Se	Zn	Hb	BMIZ	HAZ
Sex (male)	1.00													
Ethnicity (black)	0.16^1^	1.00												
Ethnicity (white)	−0.08	−0.21	1.00											
Birth order (≥3)	0.08	0.09	−0.08	1.00										
SES (extremely low)	0.05	0.05	0.05	0.15^1^	1.00									
Helminth infection	0.16^1^	0.04	−0.12^1^	0.14^1^	0.14^1^	1.00								
De-worming treatment	−0.04	0.08	−0.05	0.08	0.02	−0.10	1.00							
Maternal weight	−0.01	−0.03	0.00	0.01	−0.13^1^	0.02	0.05	1.00						
Maternal height	−0.00	−0.04	0.04	−0.22^1^	−0.04	0.00	−0.01	0.33^2^	1.00					
Serum Se	0.13^1^	−0.01	0.03	−0.06	−0.07	−0.15^1^	−0.01	−0.09	0.05	1.00				
Serum Zn	0.00	0.01	0.01	−0.11^1^	−0.01	−0.13^1^	0.09	−0.08	0.05	0.15^1^	1.00			
Haemoglobin (Hb)	0.00	0.00	0.06	−0.06	−0.11^1^	−0.15^1^	0.02	0.11	0.04	0.22^2^	0.17^1^	1.00		
BMIZ	−0.02	0.04	−0.07	−0.06	−0.20^1^	−0.03	0.07	0.28^2^	−0.05	0.05	−0.06	0.18^1^	1.00	
HAZ	−0.04	−0.06	−0.03	−0.14^1^	−0.09	−0.08	0.02	0.30^2^	0.39^2^	0.04	−0.08	0.09	0.18^1^	1.00

*SES* socio-economic status, *mat* maternal, *BMIZ* body mass index Z-score, *HAZ* height-for-age Z-score

^1^ *p* < 0.05; ^2^ *p* < 0.001

### Direct and indirect effects of SEM variables on HAZ and BMIZ

In the final SEM (Fig. 1), HAZ and BMIZ were significantly correlated with a RMSR of 0.033 indicating the model was a good fit. The coefficient of determination, akin to *R*2 in linear regression, was 0.39.

Table 4 shows the direct, indirect, and the combined total effects of each of the chosen variables on HAZ and BMIZ. Maternal height was the only variable that was directly and significantly positively associated with HAZ-scores, with the largest standardized regression weight of all the observed variables (i.e., β = 0.39). Three variables − ethnicity (being white), having a helminth infection, and serum zinc − were all directly and significantly negatively associated with HAZ. Significant indirect negative associations with HAZ-scores were sex (being male), helminth infection, and serum selenium, whereas de-worming treatment had the only indirect and significant positive association with HAZ.

**Table 4:** Direct and indirect effects of SEM variables on HAZ and BMIZ (*n* = 376)

HAZ	Direct	Indirect	Total	*p*
Sex (male)	−0.03	−0.02^1^	−0.04	0.219
Ethnicity (black)	−0.06		−0.06	0.240
Ethnicity (white)	−0.07^2^		−0.07	<0.001
Birth order	−0.06		−0.06	0.205
SES (extremely low)		−0.01	−0.01	0.073
Helminth infection	−0.09^1^	−0.01^2^	−0.09	0.014
De-worming treatment		0.01^1^	0.01	0.038
Maternal height	0.39^2^		0.39	<0.001
Serum selenium		−0.02^1^	−0.02	0.027
Serum zinc	−0.11^1^		−0.11	0.006
BMIZ	Direct	Indirect	Total	*p*
Sex (male)	−0.01	0.003	−0.01	0.832
SES (extremely low)	−0.15^1^	−0.001^1^	−0.15	0.001
Helminth infection		−0.004^2^	−0.004	<0.001
De-worming treatment		0.000	0.00	0.208
Maternal weight	0.21^2^		0.21	<0.001
Serum selenium		0.02^1^	0.02	0.004
Serum zinc	−0.06		−0.06	0.267
Haemoglobin	0.14^2^		0.14	<0.001

^1^ *p* < 0.05 ^2^ *p* < 0.001

Maternal weight and haemoglobin were both directly and significantly positively associated with BMIZ-scores, whereas the only variable with a direct and significant negative association with BMIZ-scores was an extremely low SES (Table 4). Both an extremely low SES and helminth infection were indirectly and significantly negatively associated with BMIZ-scores, whereas serum selenium had the only indirect and significant positive association with BMIZ (Table 4).

## Discussion

To our knowledge this is the first study to use a SEM to examine direct and indirect relationships between socio-economic, biological, environmental, and micronutrient variables with the potential to influence height and adiposity in disadvantaged NE Brazilian preschoolers.

### Role of biological and socio-economic variables on HAZ and BMIZ scores

Maternal height was the only unmodifiable biological variable to have a strong positive and direct effect on height in these preschoolers, even though inter-generational poverty had the potential to negatively impact on the genetic growth potential of their mothers living in such disadvantaged settings. Indeed, 40 % of the mothers with stunted or mildly stunted preschoolers had short stature, consistent with earlier reports in Northeast Brazil [18], possibly contributed by intrauterine growth retardation, and/or morbidity and malnutrition experienced during childhood [27]. Ethnicity (being white) was also an unmodifiable biological variable with a direct but negative effect on HAZ on these preschoolers [23]. No other biological or socio-economic variables had a significant effect, either direct or indirect, on HAZ score.

More than 70 % of the overweight and obese children studied here had overweight or obese mothers (Table 2), so the direct and strong positive association between maternal body weight and BMIZ-scores of the preschoolers is not unexpected [28, 29]. Some investigators suggest that maternal obesity leads to an increase in the number of adipocytes and pancreatic ß-cell hyperplasia during childhood, which in turn predisposes children to obesity and the associated long-term deleterious effects of insulin-resistance and other metabolic disorders [30, 31]. Others argue that this explanation ignores the effects of confounding factors such as SES, which in our model had both a significant direct and indirect negative association with BMIZ of the preschoolers (Table 4). Such a negative relationship is of concern because food insecure households in Brazil often rely on cheap, energy-dense, nutrient-poor foods, which can lead to excessive maternal weight gain as well as suboptimal growth during childhood [32].

### Role of micronutrient status and environmental variables on HAZ and BMIZ scores

There is accumulating evidence that selenium interacts with zinc by several mechanisms [33]. In addition, selenium and zinc each have an independent and critical role in immune competence [34, 35], which may account, in part, for the pathways linking helminth infections with serum selenium and serum zinc, and in turn with HAZ and BMIZ. For example, low selenium status has the potential to compromise zinc status, which together result in a marked reduction in immune competence, thus increasing the likelihood that infection with helminths in the preschoolers is associated with loss of appetite, diarrhoea, and malabsorption [26]. Such disturbances may have contributed to the negative effects of helminths on height and adiposity of the preschoolers studied here (Table 4), and by other investigators for children in NE Brazil [11]. Indeed, a 1 standard deviation increase in helminth infection in this study was calculated to lead to a 0.09 decrease in the HAZ expressed as standard deviation units. In addition, the unstandardized coefficients show that the presence of helminth infection reduced HAZ by 0.23 (se = 0.11) and BMIZ by 0.01 (0.002).

In our earlier report, the preschoolers, especially the boys living in the most impoverished households, were at the greatest risk for helminth infections [16], possibly because boys are more likely to play bare-footed outside. These findings are also apparent from the inter-relationships observed in our SEM (Fig. 1) between extremely low SES, being male, and helminth infections. Further, the indirect but positive relationship between treatment of helminth infections and HAZ highlight the importance of de-worming; only 50 % of the preschoolers had been treated in the past six months.

We are not the first to report a positive relationship between Hb and BMI among Brazilian children (Table 4) [36]. In our study, this effect may be linked to a larger intake of fortified iron-rich daycare meals by the heavier children. Certainly the provision of iron-rich meals in the daycares was probably responsible for the low prevalence of iron deficiency anaemia (<10 %) reported earlier among these preschoolers [17]. The negative relationship between serum zinc and both HAZ and BMIZ is not unexpected in view of the strong association between HAZ and BMIZ observed in the correlation matrix (Table 3). Investigators studying the nutrition transition in Chile reported that tallness, and to a lesser extent stunting, was associated with an increased risk of obesity in childhood [37]. This finding is attributed to increased hormonal and skeletal maturation that in turn leads to faster linear growth in childhood and earlier puberty [38].

### Strengths and limitations

The SEM has both strengths and limitations. Our model had a good fit with a RMSR of less than 0.05, and a coefficient of determination that explained a moderate amount of the total variance. The comprehensive data collected in our earlier cross-sectional study [16, 17] allowed us to test a range of both unmodifiable and modifiable variables simultaneously in our theoretical model with the potential to impact on the height and adiposity of these disadvantaged preschoolers. Surprisingly, the effect of birth order on HAZ, although negative, was not significant, possible because our criteria to define a large number of children in a family (i.e. ≥3) were too small. We recognize that our use of BMI as a measure of adiposity has some limitations, especially when applied among the differing race-ethnic groups studied here. BMI is not a precise measure of fatness, and tends to be also correlated with muscle and lean body mass, and possibly also with height within certain age groupings. Moreover, although the BMIZ-scores used here represent a measure of weight, adjusted for height, sex, and age of the preschoolers, the same BMIZ-score does not necessarily correspond to the same percentage of body fatness across the ethnic groups studied here [39]. Hence, future studies should consider employing more precise methods for measuring body fat in children such as dual-energy X-ray absorptiometry (DXA) or the BOD POD method based on air-displacement plethysmography, which have been reported to agree within 2 % body fat for children [40].

The negative relationships between helminths and HAZ and BMIZ among these preschoolers from poor communities along with the positive indirect association between de-worming treatment and HAZ have highlighted the importance of including preschool children living in at-risk environments in the World Health Organization (WHO) de-worming recommendation [41] as well as school-aged children. Nevertheless, the biological direction of the relationships implied by the direct and indirect effects in the cross-sectional model must be interpreted cautiously, as the model conveys causal assumptions and not validated conclusions. In addition, our sample was restricted to disadvantaged preschoolers attending philanthropic daycares so our findings cannot be generalized to other daycare settings for preschoolers.

## Conclusions and recommendations

Multiple variables influenced the height and body mass indices of these preschoolers from poor communities. We identified helminth infection as a significant modifiable variable adversely affecting both HAZ and BMIZ, even though the prevalence was relatively low (i.e., <20 %), highlighting that the WHO de-worming recommendation should include preschoolers as well as school-aged children living in at-risk environments. Our findings also highlight that despite on-going provision of regular physical activity, micronutrient-rich meals, clean drinking water and hygienic sanitation in the daycares, and nutrition education for the mothers, some of these preschoolers from disadvantaged households remained susceptible to both over- and under-nutrition.

## Abbreviations

SEM: Structural equation modeling
HAZ: Height-for-age Z-score
BMIZ: Body mass index Z-score
WHZ: Weight-for-height Z-score
SES: Socio-economic status
WHO: World Health Organization
BMI: Body mass index
Hb: Haemoglobin
RMSR: Root-mean-square-residual
